# Conservative management of an advanced external cervical resorption with internal approach using bio‐ceramic materials: A case report

**DOI:** 10.1002/ccr3.8378

**Published:** 2023-12-28

**Authors:** Sholeh Ghabraei, Behnam Bolhari, Nasim Hashemi, Hamidreza Gharehchahi

**Affiliations:** ^1^ Department of Endodontics, School of Dentistry Tehran University of Medical Sciences Tehran Iran; ^2^ Kerman Neuroscience Research Center Kerman University of Medical Science Kerman Iran

**Keywords:** bio‐ceramic material, dental operating microscope, endodontic treatment, external cervical resorption, internal approach, one‐beam computed tomography

## Abstract

**Key clinical message:**

A successful management of an advanced external cervical resorption using a conservative approach with CBCT, dental operating microscope, and a new bio‐ceramic material.

**Abstract:**

External cervical resorption (ECR) is a pathologic condition that is initiated on the external aspect of the root, below the epithelial attachment in the cervical position. This article will report a case of external cervical resorption (ESR) in an advanced stage, which was asymptomatic and was incidentally detected in a follow‐up radiograph after the end of orthodontic treatment. Cone‐beam computed tomography (CBCT) was prescribed to accurately diagnose the resorptive lesion and differentiate it from internal root resorption (IRR), and the final diagnosis was Heithersay's class IV ECR. Considering the health of the periodontium and the absence of attachment loss, it was decided to use a conservative internal approach to the management of this case. After the treatment, the patient was asymptomatic and the radiographic examinations showed no signs of peri‐radicular pathology during the follow‐up period. With the correct case selection and the availability of the appropriate materials and equipment such as a dental operating microscope (DOM) and bio‐ceramic materials, the internal approach can be a successful and minimally invasive treatment, even for the management of advanced ECR cases.

## Graphical abstract

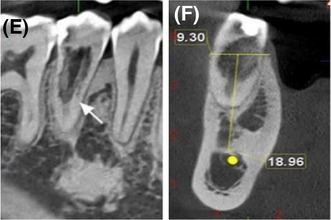

## 1 INTRODUCTION

Root resorption in permanent teeth is a pathological process resulting in a loss of dentin, cementum, and/or bone. This condition, depending on its origin, can be internal or external.^1^ ECR starts from the external aspect of the root in the cervical part of the tooth, below the epithelial attachment. The etiology and pathogenesis of ECR are not yet fully understood.^2^ However, the resorptive process is the same for ECR as it is for any other type of resorption, in such a way that damage to the protective nonmineralized layers allows the clastic cells to bind to the underlying dentine and stimulate the resorptive process.^3^ Predisposing factors for ECR include orthodontics, trauma, parafunctional habits, poor oral hygiene, malocclusion, and extraction of the neighboring tooth, respectively.^4^ In most cases, it is asymptomatic and is incidentally detected during radiographic examination. Due to the presence of granulation tissues, a pink or red discoloration may develop at the cervical region of the tooth.^5^ Probing depth may be increased if loss of periodontal attachment occurs in the region of the resorption.^6^ As the process progresses, perforation of the root canal wall and bacterial contamination of the pulp may occur. The affected tooth may develop pulpitis and the associated clinical symptoms. Pulp necrosis and chronic periapical periodontitis may eventually develop.^7^ Radiographic features of the lesion include the radiolucency that may have well‐defined or irregular margins in the cervical aspect of the tooth. However, in long‐standing lesions, with the deposition of fibro‐osseous tissues, the radiolucency may appear with a cloudy appearance.^8^ The difference between ECR and IRR, especially in cases where the ECR has resulted in perforation of the root canal wall or when the IRR has caused perforation, is very challenging and may be impossible.^2^ Two‐dimensional radiography is not sufficient to precisely detect the location and extent of the lesion and to differentiate between IRR and ECR. However, prescribing periapical radiography with different horizontal angles can be helpful.^9^ The use of CBCT can be advantageous in accurately diagnosing and differentiating between IRR and ECR, in addition, due to the important role of early detection of ECR in prognosis and treatment design the use of CBCT can be helpful.^10^ In small lesions, where the pulp is not involved and is classified as Heithersay's class 1 or 2,^11^ it can be treated with surgical access without root canal therapy using adhesive material such as Glass Ionomer cement or composite resin. Bio‐dentin may prove to be a particularly suitable material for restoring these defects because it may combine acceptable esthetics with the ability to support PDL attachment. When perforation of the root canal wall occurs, a combination of surgical access and orthograde root canal treatment or nonsurgical treatment with the internal approach for sealing the lesion can be done. For this purpose, materials such as MTA, Biodentin, CEM Cement, and cold ceramic (CC) can be used.^12^

Cold ceramic (Monsefteb, Yazd, Iran) is a mineral trioxide aggregate (MTA)‐like material with similar clinical applications to other calcium silicate cements (CSCs). CC is also biocompatible and nontoxic and has appropriate radiopacity. It is employed for various dental procedures, such as filling root ends, repairing root perforations, creating apical barriers in teeth with open apices, and possibly as a paste for abstracting root canals. Additionally, it can be used as a covering material for pulp capping and pulpotomy. The sealing ability of this material has been reported to be better than glass ionomer cement and amalgam. In the presence of moisture, cold ceramic sets initially within 15 min and reaches a complete set within 24 h.^13^, ^14^ CC showed high biocompatibility and favorable cell attachment and also induced increased expression of osteo/odontogenic differentiation markers.^15^ One study has shown that the marginal adaptation of cold ceramic is more than MTA in a long time.^16^

## 2 CASE

**FIGURE 1:**
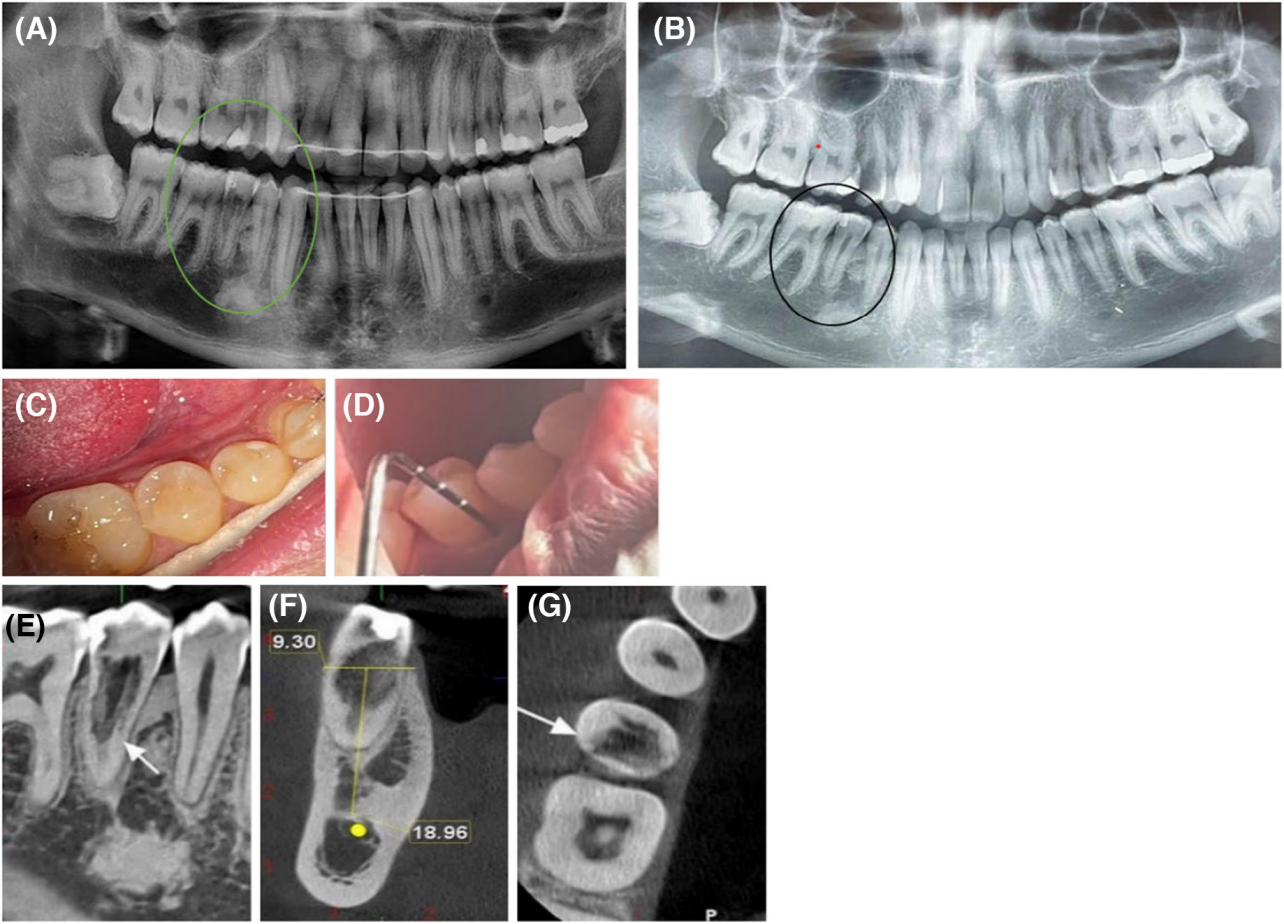
(A) panoramic radiograph after the end of orthodontic treatment. (B) Panoramic radiograph before orthodontic treatment, (C) intraoral examination, (D) probing depth, (E) periapical radiograph, (F) CBCT, coronal view, and (G) CBCT, axial view.

**FIGURE 2:**
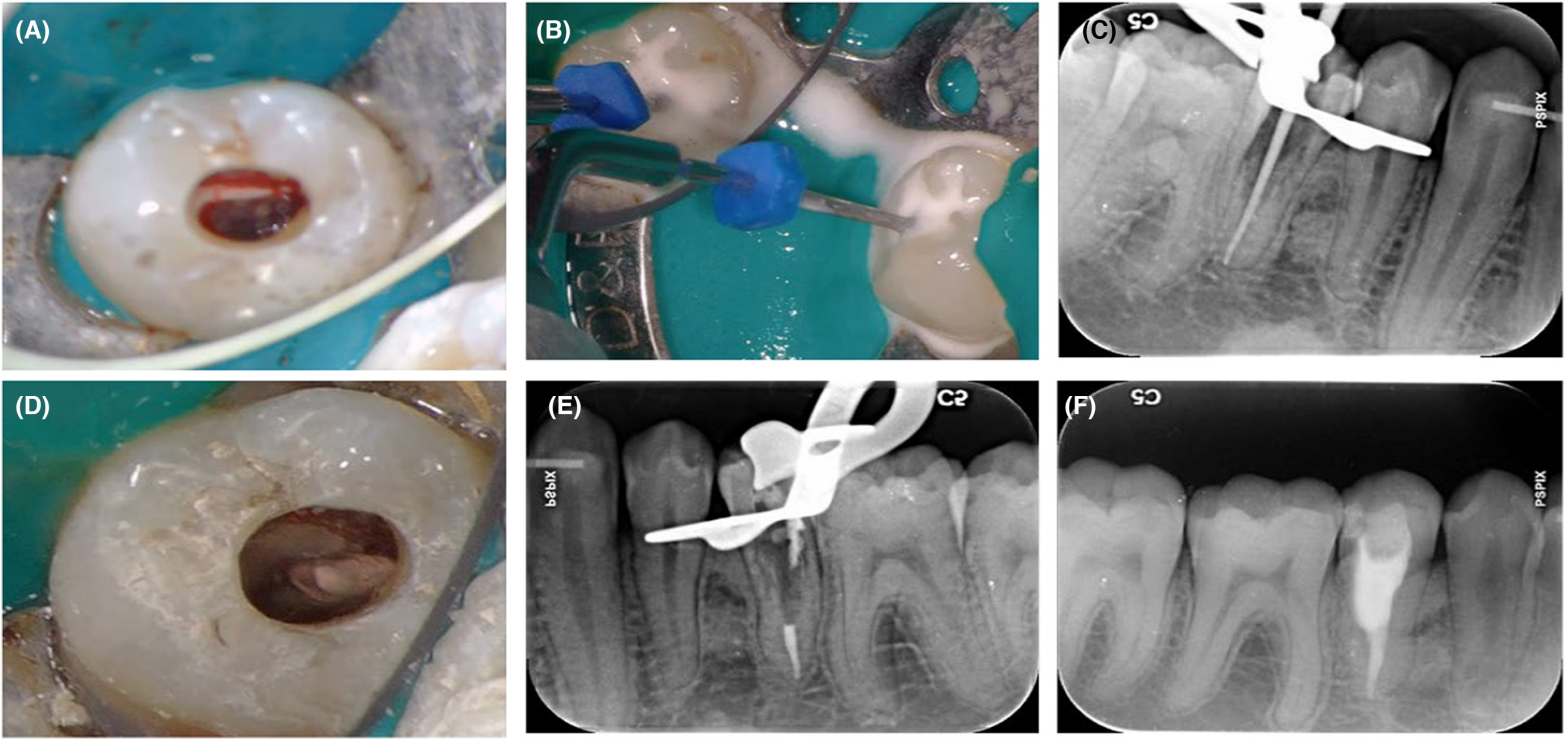
(A) Conservative access cavity preparation. (B) Sodium hypochlorite solution and its activation with ultrasonic. (C) Confirming MAC by radiography. (D) Cutting off the gutta percha at the 3 mm area from the apex under DOM. (E) Cutting off the gutta percha at the 3 mm area from the apex. (F) The remaining portion of the canal was filled with cold ceramic.

**FIGURE 3:**
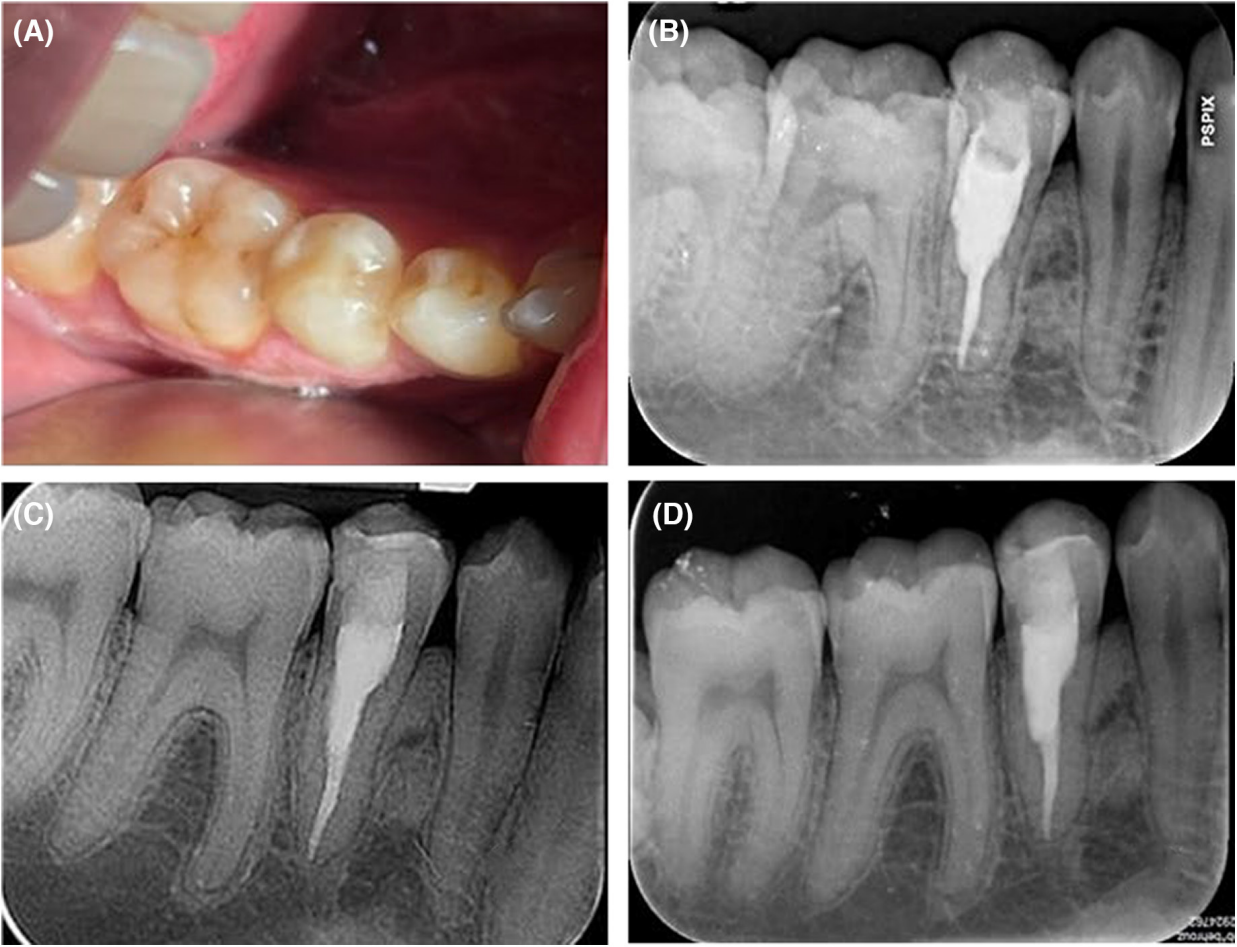
(A) Tooth was restored with composite resin adhesive. (B) 3‐month follow‐up, (C) 6‐month follow‐up, (D) 20‐month follow‐up.

A 28‐year‐old healthy male patient, after taking a panoramic radiograph for a follow‐up examination after the end of orthodontic treatment, was referred by his dentist to the endodontic department of the Faculty of Dentistry of Tehran University of Medical Sciences with concerns about his right mandibular second premolar with a radiolucency inside the root canal. The patient had undergone orthodontic treatment within the last 2 years and currently wore a retainer. Radiographs taken before orthodontic treatment did not reveal any radiolucency in this tooth (Figure 1A), but in the recent panoramic radiograph, a resorptive lesion was visibly observed (Figure 1B). The intraoral examination did not reveal any discoloration, pain, or swelling (Figure 1C). The probing depth was within a normal limit (Figure 1D). Further tests showed that the tooth was nonresponsive to thermal electric pulp tests, so the diagnosis for this tooth was pulpal necrosis and normal periapical tissue. The periapical radiograph revealed an irregular radiolucency in the cervical and middle third of the root canal area (Figure 1E). A CBCT was recommended to better define and visualize the lesion (Figure 1F,G). Based on the clinical and radiographic findings, the lesion was diagnosed as an ECR that initiated from disto‐lingual surfaces and spread into the root canal. After discussing the situation with the patient, we proposed a treatment plan including nonsurgical root canal therapy and an internal orthograde approach with cold ceramic to repair the resorptive defect. The patient was informed about the treatment plan and provided consent to proceed. At the first visit, local anesthesia (xylopen2%, Exsir, Iran) was administered and a conservative access cavity was prepared (Figure 2A). The tooth was isolated with a rubber dam and granulation tissue was noted along with excessive bleeding within the canal. Following the use of a 2.5% sodium hypochlorite solution and its activation with ultrasonic (Figure 2B), the working length of the root canal was established at 20 mm, and the root canal was completely prepared with the crown down technique using rotary NiTi instruments (DENCO Super Files III, Longhua, Shenzhen, China) with the aid of magnification under Dental operating microscope (DOM) (OPMI Pico Zeiss Dental Microscope, Germany). Granulation tissue was carefully removed from within the root canal, and bleeding was controlled. Next, a calcium hydroxide dressing was placed within the canal, and the access cavity was restored with temporary cement (Morvabon Z.O.E Cement, Iran). At the patient's second visit, 1 week after the initial appointment, the temporary filling was removed and the affected tooth was isolated with a rubber dam. Under magnification with DOM, the calcium hydroxide was carefully removed. The canal was then irrigated with 2.5% hypochlorite solution and subsequently dried. A master apical cone (MAC) with a size of 35 (6%) was then inserted into the working length of the canal and confirmed by radiography (Figure 2C). The MAC was placed in the canal using ENDO Seal MTA sealer (Marruci EndoSeal MTA, USA) and was cut off with a heat carrier at the 3 mm from the apex (Figure 2D,E). Following root canal therapy, the remaining portion of the canal was filled with cold ceramic (Monsefteb, Yazd, Iran) (Figure 2F). Subsequently, a moist cotton was placed over the MTA to facilitate proper setting, and the temporary restoration was placed. During the third visit, which occurred 2 days later, the MTA setting was evaluated, and the patient was referred for tooth restoration. The affected tooth was restored using a composite resin adhesive (Figure 3A).

Follow‐Up: During the 3‐, 6‐, and 20‐month follow‐up visits (Figure 3B–D), the patient reported no complications. Although the crown of the affected tooth exhibited slight discoloration, it was negligibly perceptible since it was beyond the aesthetic zone. Radiographically, the apical region appeared to be healthy, with no changes in the surrounding bone.

## 3 DISCUSSION

The etiology of ECR is not yet fully understood. However, several studies have identified orthodontic treatment as a potential etiological factor in the development of ECR.^4^, ^17^, ^18^, ^19^

In this case, the patient had undergone orthodontic treatment in the past, and radiographs taken before orthodontic treatment did not reveal any resorptive lesion. However, in the follow‐up radiograph after orthodontic treatment, a significant resorptive lesion was present in the affected tooth. Therefore, in this case, it can be said that orthodontics has been a predisposing factor for ECR.

Managing ECR is a challenging task in endodontics. The first step in managing ECR is to make an accurate diagnosis and determine the prognosis of the affected tooth.^20^

One of the diagnostic challenges in this case was differentiating between an external cervical root resorption and an internal root resorption. It is imperative to differentiate IRR from ECR.

An IRR with perforation in the cervical area of the root could also have a similar appearance to ECR.^21^ Two‐dimensional radiography may not always differentiate these cases and the use of CBCT can be highly beneficial.^22^

In our case, performing CBCT before intervention enabled us to identify the nature, location, and extent of the resorptive lesion. The information obtained through CBCT greatly enhanced our ability to manage these cases. Therefore, based on the location and pattern of resorption, the final diagnosis was Heithersay's class IV ECR.

Managing teeth with ECR that have an uncertain prognosis represents a significant challenge for many endodontists. In such cases, tooth extraction and implant replacement may represent an alternate and predictable treatment modality. However, it is essential to consider variables such as the location of the affected tooth, its proximity to structures such as nerves and sinuses, the quality and quantity of available bone, the patient's systemic health status, their socioeconomic status, motivation, and potential complications of surgery.^23^

Numerous therapeutic approaches are available for addressing external cervical root resorption. Selecting a treatment method hinges on the particular instance and the cause of the resorption. The chief objective of any treatment is to thoroughly eliminate the resorptive tissue, arrest the resorptive process and seal the compromised area.^24^, ^25^

In this case, since the periodontium was healthy and no bone defect was observed in the resorptive area, it was decided to remove the resorptive tissues and seal the defective area using an internal approach because accessing the lesion surgically would necessitate the removal of healthy bone that will subsequently lead to gingival recession.^26^

The preparation of a conservative access cavity is essential for minimal removal of the tooth structure.^27^

The use of DOM is very helpful for accurately observing the perforation site, completely removing the resorptive tissues, and treating ECR with internal approach, it is useful too.^28^

In our case, the resorption area was fully visible under DOM. Thus, using an internal approach, we could eliminate the resorptive tissue and completely seal the resorption area with a bio‐ceramic material.

The apical third area of these teeth, where there was no resorption or perforation was filled with gutta‐percha and a bio‐ceramic sealer to better control the working length.^29^ Then we filled the rest of the canal with cold ceramic.

Cold ceramic has a good consistency after mixing, good handling properties and it is nontoxic and biocompatible with host tissues.^13^

Based on the results obtained from the studies, the sealing property of CC is better than MTA in blood‐contaminated conditions and similar to MTA in dry and saliva‐contaminated conditions and setting time of CC is about 15 min, which is shorter than MTA, was reported as about 165 min, also there was no significant difference in the average tooth discoloration between cold ceramic and MTA.^13^

After the treatment, the patient was asymptomatic and the radiographic examinations showed no signs of peri‐radicular pathology during the follow‐up period.

As a limitation of our study, we had a short follow‐up time, and we plan to report longer follow‐up findings when they become available. Further research on the etiology and long‐term outcome of this treatment plan is also needed.

Because accessing and removing the resorption areas through the internal approach is difficult, long‐term follow‐up is necessary to ensure there is no recurrence of the resorption. If there is evidence of resorption, it may be necessary to consider the external surgical gingival flap approach.^30^

Using CBCT for the initial evaluation and diagnosis of resorption, in addition to two‐dimensional radiography, is essential. However, intraoral periapical radiographs are suitable for continuous follow‐up in accordance with ALARA principles, and usually, there is no need to prescribe CBCT.^31^

## 4 CONCLUSION

In cases where the external root resorption is limited to the tooth and there is no bone defect and attachment loss, the internal approach can be a conservative method for ECR management. Using this method, it is possible to remove the resorptive tissues and seal the defect area without surgery. However, this treatment requires the use of magnification and direct observation of the resorption area.

## AUTHOR CONTRIBUTIONS

**Sholeh Ghabraei:** Conceptualization; investigation; methodology; supervision. **Behnam Bolhari:** Data curation; investigation; writing – original draft. **Nasim Hashemi:** Investigation; methodology; project administration; writing – review and editing. **Hamidreza Gharehchahi:** Data curation; formal analysis; investigation.

## FUNDING INFORMATION

The study had no financial support.

## ETHICS STATEMENT

This study was conducted according to the guidelines laid down in the Declaration of Helsinki and all procedures involving research study participants were approved by the Ethics Committee of Tehran University of Medical Sciences (TUMS).

## CONSENT

Written informed consent was obtained from the patient to publish this report in accordance with the journal's patient consent policy.

## Data Availability

The data that support the findings of this study are available on request from the corresponding author. The data are not publicly available due to privacy or ethical restrictions.

## References

[1] Patel S , Saberi N , Pimental T , Teng PH . Present status and future directions: root resorption. Int Endod J. 2022;55(Suppl 4):892‐921. doi:10.1111/iej.13715 35229320 PMC9790676

[2] Patel S , Kanagasingam S , Ford TP . External cervical resorption: a review. J Endod. 2009;35(5):616‐625. doi:10.1016/j.joen.2009.01.015 19410071

[3] Lewusz‐Butkiewicz K , Kaczor‐Wiankowska K , Kulas‐Bałaban K‐W , Szmidt‐Kądys M . Treatment of external cervical resorption and its late complication: a case report. Iran Endod J. 2022;17(1):48‐51. doi:10.22037/iej.v17i1.36672 36703870 PMC9868990

[4] Kandalgaonkar SD , Gharat LA , Tupsakhare SD , Gabhane MH . Invasive cervical resorption: a review. J Int Oral Health. 2013;5(6):124‐130. PMC3895730 24453457

[5] Heithersay GS . Clinical, radiologic, and histopathologic features of invasive cervical resorption. Quintessence Int. 1999;30(1):27‐37. 10323156

[6] Gijón VR , Martín CL , Encinas RMP , Navajas JM . Aetiological, histopathological, clinical, diagnostic and Therapeutical features of idiopathic cervical resorption. Dent Update. 2016;43(10):964‐970. doi:10.12968/denu.2016.43.10.964 29155538

[7] Ricucci D , Milovidova I , Amantea M , Girone C , Rôças IN , Siqueira JF Jr . Histologic features of external cervical resorption affecting impacted maxillary canines: a report of 2 cases. J Endod. 2023;49(6):720‐729. doi:10.1016/j.joen.2023.03.012 37001728

[8] Luso S , Luder HU . Resorption pattern and radiographic diagnosis of invasive cervical resorption. A correlative microCT, scanning electron and light microscopic evaluation of a case series. Schweiz Monatsschr Zahnmed. 2012;122(10):914‐930. doi:10.5167/uzh-65911 23097140

[9] Vaz de Souza D , Schirru E , Mannocci F , Foschi F , Patel S . External cervical resorption: a comparison of the diagnostic efficacy using 2 different cone‐beam computed tomographic units and periapical radiographs. J Endod. 2017;43(1):121‐125. doi:10.1016/j.joen.2016.09.008 27939734

[10] Talpos‐Niculescu RM , Nica LM , Popa M , Talpos‐Niculescu S , Rusu LC . External cervical resorption: radiological diagnosis and literature (review). Exp Ther Med. 2021;22(4):1065. doi:10.3892/etm.2021.10499 34434279 PMC8353645

[11] Heithersay GS . Invasive cervical resorption following trauma. Aust Endod J. 1999;25(2):79‐85. doi:10.1111/j.1747-4477.1999.tb00094.x 11411085

[12] Arıcan B , Sesen Uslu Y , Sarıalioğlu GA . Resistance to fracture of simulated external cervical resorption cavities repaired with different materials. Aust Endod J. 2023;49(1):174‐182. doi:10.1111/aej.12714 36354094

[13] Modaresi J , Hemati HR . The cold ceramic material. Dent Res J (Isfahan). 2018;15(2):85‐88. 29576770 PMC5858076

[14] Modaresi J , Parashos P , Mousavi R , Mirzaeeian A , Almodaresi Z . Treatment of strip perforation using cold ceramic. Dent Res J (Isfahan). 2023;20:31. 37180691 PMC10166746

[15] Khedmat S , Sarraf P , Seyedjafari E , Sanaei‐rad P , Noori F . Comparative evaluation of the effect of cold ceramic and MTA‐angelus on cell viability, attachment and differentiation of dental pulp stem cells and periodontal ligament fibroblasts: an in vitro study. BMC Oral Health. 2021;21:628. doi:10.1186/s12903-021-01979-1 34876089 PMC8650362

[16] Mokhtari F , Modaresi J , Javadi G , Davoudi A , Badrian H . Comparing the marginal adaptation of cold ceramic and mineral trioxide aggregate by means of scanning electron microscope: an in vitro study. J Int Oral Health. 2015;7(9):7‐10. PMC4589723 26435608

[17] Chen Y , Huang Y , Deng X . External cervical resorption‐a review of pathogenesis and potential predisposing factors. Int J Oral Sci. 2021;13(1):19. doi:10.1038/s41368-021-00121-9 34112752 PMC8192751

[18] Discacciati JA , de Souza EL , Costa SC , Sander HH , Barros Vde M , Vasconcellos WA . Invasive cervical resorption: etiology, diagnosis, classification and treatment. J Contemp Dent Pract. 2012;13(5):723‐728. doi:10.5005/jp-journals-10024-1217 23250183

[19] Irinakis E , Aleksejuniene J , Shen Y , Haapasalo M . External cervical resorption: a retrospective case‐control study. J Endod. 2020;46(10):1420‐1427. doi:10.1016/j.joen.2020.05.021 32525057

[20] Patel J , Beddis HP . How to assess and manage external cervical resorption. Br Dent J. 2019;227(8):695‐701. doi:10.1038/s41415-019-0781-x 31654002

[21] Heboyan A , Avetisyan A , Karobari MI , et al. Tooth root resorption: a review. Sci Prog. 2022;105(3):368504221109217. doi:10.1177/00368504221109217 35759366 PMC10358711

[22] Bhuva B , Barnes JJ , Patel S . The use of limited cone beam computed tomography in the diagnosis and management of a case of perforating internal root resorption. Int Endod J. 2011;44(8):777‐786. doi:10.1111/j.1365-2591.2011.01870.x 21371054

[23] Bhattacharyya S , Das DP , Bhattacharyya A , Maity AB , Das D . Clinical guideline and treatment planning decisions of single‐tooth implants versus preserving natural teeth with nonsurgical endodontic therapy. J Family Med Prim Care. 2020;9(6):2654‐2658. doi:10.4103/jfmpc.jfmpc_128_20 32984102 PMC7491817

[24] Eftekhar L , Ashraf H , Jabbari S . Management of Invasive Cervical Root Resorption in a mandibular canine using biodentine as a restorative material: a case report. Iran Endod J. 2017;12(3):386‐389. doi:10.22037/iej.v12i3.16668 28808471 PMC5527220

[25] Fonsêca KS , Romano CMR , Xavier JMB , Scardini IL , Ribeiro FC . Management of external cervical resorption with internal approach: 18 months of follow‐up. Res Soc Dev. 2023;12(6):e17912642184. doi:10.33448/rsd-v12i6.42184

[26] Zucchelli G , De Sanctis M . A novel approach to minimizing gingival recession in the treatment of vertical bony defects. J Periodontol. 2008;79(3):567‐574. doi:10.1902/jop.2008.070315 18315442

[27] Nilsson E , Bonte E , Bayet F , Lasfargues JJ . Management of internal root resorption on permanent teeth. Int J Dent. 2013;2013:929486. doi:10.1155/2013/929486 24348560 PMC3857824

[28] Suehara M , Sano Y , Sako R , et al. Microscopic endodontics in infected root canal with calcified structure: a case report. Bull Tokyo Dent Coll. 2015;56(3):169‐175. doi:10.2209/tdcpublication.56.169 26370577

[29] Jawami A , Soo E . Iatrogenic extrusion of calcium silicate cements on teeth associated with large periapical lesion: a case report with 12‐month follow‐up. J Dent Indones. 2022;29(2):154‐159. doi:10.14693/jdi.v29i2.1252

[30] Chen Y , Huang Y , Deng X . A review of external cervical resorption. J Endod. 2021;47(6):883‐894. doi:10.1016/j.joen.2021.03.004 33745945

[31] Bardini G , Orrù C , Ideo F , Nagendrababu V , Dummer P , Cotti E . Clinical management of external cervical resorption: a systematic review. Aust Endod J. 2023;49(1):174‐182. doi:10.1111/aej.12794 37702252

